# A genetic variant in the *APE1/Ref-1 *gene promoter -141T/G may modulate risk of glioblastoma in a Chinese Han population

**DOI:** 10.1186/1471-2407-11-104

**Published:** 2011-03-23

**Authors:** Keke Zhou, Dezhi Hu, Juan Lu, Weiwei Fan, Hongliang Liu, Hongyan Chen, Gong Chen, Qingyi Wei, Guhong Du, Ying Mao, Daru Lu, Liangfu Zhou

**Affiliations:** 1 Neurosurgery Department of Huashan Hospital, Fudan University, Shanghai, PR China; 2 State Key Laboratory of Genetic Engineering and MOE Key Laboratory of Contemporary Anthropology, School of Life Sciences and Institutes for Biomedical Sciences, Fudan University, Shanghai, PR China; 3 Department of Epidemiology, The University of Texas M.D. Anderson Cancer Center, Houston, TX, USA; 4 Neurosurgery Department of People's Hospital of Putuo District, Shanghai, PR China

**Keywords:** DNA repair, glioma, APE1/Ref-1, association study, false positive report probability

## Abstract

**Background:**

The human apurinic/apyrimidinic endonuclease 1/Redox effector factor-1 (*APE1/Ref-1*) is implicated in tumor development and progression. Recently, the *APE1/Ref-1 *promoter -141T/G variant (rs1760944) has been reported to be associated with lung cancer risk. Given the importance of *APE1/Ref-1 *in both DNA repair and redox activity, we speculate that the -141T/G polymorphism may confer individual susceptibility to gliomas or its subtypes.

**Methods:**

The *APE1/Ref-1 *-141T/G polymorphism was analyzed in a case-control study including 766 glioma patients (among them 241 glioblastoma, 284 astrocytomas except for glioblastoma and 241 other gliomas) and 824 cancer-free controls from eastern China. Genotyping was performed with Sequenom MassARRAY iPLEX platform by use of allele-specific MALDI-TOF mass spectrometry assay. We estimated odds ratios (ORs) and 95% confidence intervals (95% CIs) using unconditional logistic regression. A test of trend was calculated using the genotype as an ordinal variable in the regression model. For each statistically significant association identified, we estimated the false positive reporting probability (FPRP). FPRP values less than 0.2 were consider to indicate robust associations.

**Results:**

The significant association between the *APE1/Ref-1 *promoter -141T/G polymorphism and glioma risk was not observed. However, the stratified analysis by histology revealed the variant allele G significantly decreased glioblastoma risk (OR = 0.80, 95% CI = 0.65-0.98, *P *= 0.032). Individuals with the homozygous -141GG genotype exhibited 46% reduced risk of glioblastoma (adjusted OR = 0.54, 95% CI 0.34-0.87, *P *= 0.012), compared with the TT homozygote. This result remained robust given the prior probabilities of 25% (FPRP = 0.052) and 10% (FPRP = 0.140), but not with a prior probability of 1% (FPRP = 0.643). The *P-*associated with the trend test was 0.014.

**Conclusions:**

Our results suggest that a specific genetic variant located in the *APE1/Ref-1 *promoter may modulate risk of glioblastoma, but not for other histological gliomas. Larger studies with more *APE1 *polymorphisms are required to validate these preliminary findings.

## Background

Gliomas present a group of primary brain tumors that originate from glia (from the Greek for "glue"), accounting for more than 40% of the newly diagnosed brain tumors [1]. In accordance with their clinicopathological features, gliomas are generally classified into astrocytomas, oligodendrogliomas, mixed oligoastrocytomas and ependymomas [2]. Glioblastoma multiforme (GBM), a subtype of gliomas, is the most common and the most malignant glioma (World Health Organization [WHO] grade IV) with median survival of only 12-15 months under the current standard of care [3]. Except high-dose ionizing radiation (IR), the etiology of sporadic gliomas is still poorly understood [2]. However, only a small proportion of exposed individuals will develop gliomas, suggesting a genetic predisposition of gliomas.

Exposure to IR can result in various types of DNA damage that cause cells to turn cancerous. Cells respond to this damage through the activation of various DNA repair pathways, and each pathway involves numerous molecules. The human apurinic/apyrimidinic endonuclease 1/Redox effector factor-1 (*APE1/Ref-1*) plays a central role in the base excision repair pathway for the repair of DNA damage generated by alkylation, oxidatation and IR [4]. As an endonuclease, *APE1/Ref-1 *hydrolyzes the phosphodiester bond 5' to abasic sites, leaving a normal 3'-hydroxyl group and 5'-deoxyribose phosphate terminus for DNA repair synthesis [5-7]. In addition to AP endonuclease activity, *APE1/Ref-1 *has 3'-repir diesterase or phosphatase activity [8], which is important in repairing DNA damage caused by radiation. Furthermore, *APE1/Ref-1 *also possesses a 3'-5' exonuclease activity, reported to play a role in the excision of deoxyribonucleoside analogs from DNA [9,10]. Besides its role in DNA repair, *APE1Ref-1 *also functions as a redox activator of numerous cellular transcription factors, such as AP-1, NF-кB, p53, Egr-1, c-Myb, HIF-1α, HLF, and Pax-8, that are thought to be important in carcinogenesis [11]. Knocking out *APE1/Ref-1 *in mice causes postimplanatation embryonic lethality [12,13] and any attempt to isolate stable *APE1/Ref-1 *- knockout cell lines has been so far unsuccessful, clearly indicating that the importance of its function to cell survival and propagation.

The Human *APE1/Ref-1 *is polymorphic. The most frequently evaluated variant in relation to cancer risk is the single nucleotide polymorphism (SNP) at codon 148 of exon 5 of the *APE1/Ref-1 *gene (i.e. Asp148Glu; D148E; rs1130409). Although the *APE1/Ref-1*Asp148Glu polymorphism doses not result in reduced endonuclease activity [14], the Glu allele may have higher sensitivity to IR [15]. Genetic epidemiologic studies have showed that the *APE1/Ref-1 *Glu allele alone was associated with an increased risk of breast caner [16] and gastric cancer [17], and with a decreased risk of cutaneous melanoma [18] and gliomas [19], whereas no associations were found in prostate cancer [20], pancreatic cancer [21] and glioblastoma [22]. For lung cancer, Glu allele homozygote had an increased risk in one small study [23], but no association was observed in other six larger studies [24-29]. Other *APE1/Ref-1 *polymorphisms have also been evaluated for altered cancer susceptibility. One study reported that the non-synonymous SNP Ile64Val (rs2307486) was associated with a decreased risk of non-small cell lung cancer (NSCLC) [27]. Another amino acid substitutive variant, Gln51His (rs1048945), was evaluated in two studies; no association was found for either prostate cancer [20] or lung cancer [27]. Moreover, the polymorphisms in the *APE1/Ref-1 *gene and other DNA repair genes may interact to have joint effects on risk of certain cancers [18-21,23-28].

Notably, these previous studies mainly focused on the non-synonymous SNPs of the *APE1/Ref-1 *gene. The role of *APE1/Ref-1 *promoter polymorphisms in cancer risk was little studied. Recently, the -141T/G variant (rs1760944) in the *APE1/Ref-1 *promoter has been reported to be associated with a decreased risk of lung cancer in Chinese populations [28,29]. In view of the importance of *APE1/Ref-1 *in both DNA repair and redox activity, we speculate that *APE1/Ref-1 *-141T/G variant may also confer individual susceptibility to gliomas or its certain subtypes. To test this hypothesis, the *APE1 *promoter polymorphism was investigated in a hospital-based case-control study with 766 glioma patients and 824 cancer-free controls from eastern China. Odds ratios (OR) were determined for all study patients and for those with histological subtypes of glioblastoma (241 cases), astrocytomas except for glioblastoma (284 cases), and other gliomas (241 cases).

## Methods

### Study population

The current study included 766 glioma patients and 824 healthy cancer-free controls. According to histological diagnosis, the glioma patients were stratified into three subgroups: 241 glioblastomas, 284 astrocytomas except for glioblastoma and 241 other gliomas. The subject enrollment commenced in October 2004 and is ongoing. All newly diagnosed glioma cases were inpatients for tumor resection at the Neurosurgery Department of Huashan Hospital of Fudan University, Shanghai, China. This hospital is the biggest center of neurosurgery in the east of China [30]. Histopathological diagnoses of potentially eligible cases were carried out on the basis of the biopsies or resected specimens in the Department of Neuropathology of the same hospital. Although there were no restrictions on age, gender and histology, patients who had previous cancers and/or previous radiotherapy or chemotherapy were excluded. Cancer-free control subjects were randomly recruited from annual checkup visitors (80%) and trauma outpatients (20%) at the same hospital during the similar time period and were frequency- matched to cases by gender, age (±5 years) and residence (urban or rural). The included controls self-reported no history of malignancies, genetic neurologic disorders and radiotherapy/chemotherapy. No evidence of demographic differences was detected between these trauma outpatients and the annual checkup subjects (data not shown). All participants were genetically unrelated ethnic Han Chinese people from Shanghai and its surrounding regions including Jiangsu, Zhejiang, and Anhui provinces.

Each eligible subject was face-to-face interviewed by a trained personnel who was blinded to the case-control status with a shortened structured questionnaire, which was adapted from the original version provided by Dr. Melissa Bondy for brain study in the Department of Epidemiology at M.D. Anderson Cancer center, to obtain data on demographic factors, smoking status, radiation exposure, family history of cancer and other factors. Each subject provided the informed consent and donated 3-5 ml of peripheral blood. As a result, DNA samples and questionnaires were available from 766 case and 824 control subjects representing an 85.7% and 79.2% of all eligible case and control subjects, respectively. The research protocol was approved by the Ethics Committee for Human Subject Research of Fudan University.

### Genotyping

Genomic DNA was extracted from whole-blood samples using the Qiagen Blood Kit (Qiagen, Chatsworth, CA, USA), according to the manufacturer's instructions. After extraction, genomic DNA was diluted to a final concentration of 15-20 ng/μl for the genotyping assays. Polymorphism spanning fragments were amplified by the polymerase chain reaction (PCR) and performed genotyping with Sequenom MassARRAY iPLEX platform by use of allele-specific MALDI-TOF mass spectrometry assay [31]. Primers for amplification and extension reactions were designed using MassARRAY Assay Design Version 3.1 software (Sequenom, San Diego, CA). And SNP genotypes were obtained according to the iPLEX protocol provided by manufacturer. Genotyping quality was examined by a detailed QC procedure consisting of > 95% successful call rate, duplicate calling of genotypes, internal positive control samples and Hardy-Weinberg Equilibrium (HWE) testing.

### Statistical analysis

Differences in demographic variables, smoking status, family history of cancer, and grouped allele and genotype frequencies between cases and controls were compared by the χ^2 ^test (*df *= 2). The Hardy-Weinberg Equilibrium was determined using the standard χ^2 ^test (*df *= 1) to compare the observed frequency with the expected frequency in controls. To estimate the association between the *APE1/Ref-1 *-141T/G variant and risk of gliomas, odds ratios and 95% confidence intervals (95% CI) were calculated using unconditional logistic regression with adjustment for age and gender. The homozygosity with the more frequent allele among controls was set as the reference group. A test of trend was calculated by treating the three genotypes (major allele homozygous, heterozygous and variant allele homozygous) as ordinal variables in the regression models. Furthermore, we performed stratified analyses for the *APE1/Ref-1 *-141T/G polymorphism by age (two groups: < 18 years and > 18 years), gender, smoking status, family history of cancer and histologic type. The SPSS 15.0 software (SPSS, Chicago, IL, USA) was used for all statistical analyses. All *P *values presented were two sided, and a level of *P *< 0.05 was considered statistically significant.

To further assess the probability of a spurious association due to multiple testing, we calculate the false-positive report probability (FPRP) for a SNP from an estimated OR and 95% CIs using the methodology described by Wacholder et al [32]. To assign a prior probability for the *APE1/Ref-1 *-141T/G variant, we considered that the genetic variant has been previously shown to influence expression of the *APE1/Ref-1 *gene [28,29] and has been previously reported to be associated with lung cancer risk [28,29]. In the light of this, prior probabilities of 25% (and 10%) were assigned. However, no previous studies have been reported on the association between the *APE1/Ref-1 *promoter polymorphism and glioma risk. Hence, we selected more conservative prior probabilities, 1% and 10%. In accordance with Wacholder et al [32], a standard FPRP cut-off of < 0.5 was selected with a cut-off of < 0.2 being considered more stringent.

## Results

### Characteristics of study population

The details of selected characteristics of 766 glioma cases ad 824 control subjects have been shown in Table 1. The differences in the distributions of age, gender and smoking status between cases and controls were not statistically significant (*P *= 0.353, 0.919, and 0.535, respectively). The mean age of cases (42.4 years) at diagnosis was slightly older than the reference age for controls (41.5 years). 59.1% cases and 59.9% controls were male. As shown in the previous study, the cases were more likely to report a history of cancer in first-degree relatives than controls (20.3% *vs. *15.5%; *P *= 0.027). Among 766 glioma cases, 241 were glioblastoma, 284 astrocytomas except for glioblastoma and 241 other gliomas (Table 1).

**Table 1:** Frequency distribution of selected characteristics of study subjects by the case-control status

Variable	Controls (n = 824)	Cases (n = 766)	*P *value^a^
		
	No. (%)	No. (%)	
Age (mean ± SD) (y)	41.5 ± 18.4	42.2 ± 16.0	0.439
Age (y)			0.353
Children(≤18)	60(7.3)	66(8.6)	
Adults(>18)	764(92.7)	700(91.4)	
Gender			0.919
Male	490(59.5)	453(59.1)	
Female	334(40.5)	313(40.9)	
Smoking status			0.535
Never	490(60.6)	394(62.0)	
Former	120(14.8)	81(12.8)	
Current	199(24.6)	160(25.2)	
Family history of cancer			
No	626(84.5)	496(79.7)	0.027
Yes	115(15.5)	126(20.3)	
Histology			
Glioblastoma	241(31.5)		
Astrocytomas^b^	284(37.0)		
Other gliomas^c^	241(31.5)		

^a^Two-sided χ^2 ^test.

^b^Astrocytomas including diffuse astrocytomas, anaplastic astrocytomas and other astrocytomas.

^c^Other gliomas including oligodendrogliomas, enpendymomas, or mixed glioma.

The genotyping success rate was 98.5%. The observed G allele frequency for rs1760944 T/G SNP in controls was 45.0%, which was similar to that reported in HapMap database for Chinese Han population. Genotype frequencies of rs1760944 T/G SNP among the controls did not differ significantly from those expected under HWE (*P *= 0.262). Because there was no difference in allele and genotype frequency distribution between two control groups, "annual check-up subjects" and "trauma outpatients" (data not shown), we combined these two control groups for subsequent analyses.

### *APE1/Ref-1 *rs1760944 and risk of gliomas

Allele and genotype frequencies and associated ORs (95% CI) for glioma cases and controls are presented in Table 2. The variant allele G was 45.0% in controls and 42.8% in cases. The frequencies of the TT, TG and GG genotypes of *APE1/Ref-1 *rs1760944 were 29.0%, 52.0% and19.0% in controls, and 30.4%, 51.2% and 16.3% in cases, respectively. No significant differences were found in allele and genotype distributions of *APE1Ref-1 *rs1760944 T/G between glioma cases and controls (*P *= 0.220 and 0.410, respectively). In logistic regression analysis, neither the homozygous -141GG nor heterozygous TG genotype was associated with risk of gliomas in overall population. Further analysis showed that rs1760944 T/G genotypes were not associated with gender, age, smoking status, or family history of cancer (Table 3).

**Table 2:** Analysis of association between *APE1/Ref-1 *rs1760944 and risk of all gliomas

rs1760944	Controls (824)	All Gliomas (766)			
		
	No. (%)	No. (%)	*P*^a^	OR (95% CI) ^b^	*P*^c^
Alleles					
T	898 (55.0)	858 (57.2)	0.220	1.00 (reference)	0.220
G	734 (45.0)	642 (42.8)		0.91 (0.79-1.05)	
Genotypes					
TT	237 (29.0)	233 (30.4)	0.410	1.00 (reference)	
TG	424 (52.0)	392 (51.2)		0.94 (0.75-1.18)	0.588
GG	155 (19.0)	125 (16.3)		0.81 (0.61-1.10)	0.183
			*P*^d ^for trend = 0.199		

^a^Allele and genotype frequencies in cases and controls were compared using χ^2 ^test.

^b^Allele-specific OR was not adjusted; genotype-specific ORs were adjusted for age and gender.

^c^*P *value from unconditional logistic regression analyses.

^*d*^Trend test was calculated using the genotype as an ordinal variable in the regression model.

**Table 3:** Stratified analysis of associations between APE1/Ref-1 rs1760944 genotypes and risk of all gliomas by age, gender, smoking status, and family history of cancer

Variable	Cases^a^/Controls (766/824)			Adjusted OR (95% CI)^b^	
	
	TT	TG	GG	TG versus TT	GG versus TT
Age (years)					
≤18	19/15	28/38	9/7	0.58 (0.26-1.32)	1.12
>18	214/222	364/386	116/148	0.97 (0.77-1.29)	0.80 (0.59-1.10)
Gender					
Male	133/150	233/241	76/92	1.09 (0.81-1.46)	0.63 (0.47-0.85)
Female	100/87	159/183	49/63	0.72 (0.47-1.09)	0.93 (0.63-1.37)
Smoking status					
Non-smokers	122/140	202/248	61/94	0.91 (0.67-1.25)	0.74 (0.50-1.12)
Former smokers	23/32	39/66	13/22	0.82 (0.41-1.62)	0.77 (0.32-1.87)
Current smokers	45/61	93/100	24/37	1.25 (0.77-2.03)	0.87 (0.46-1.67)
Family history of cancer					
No	156/175	250/323	81/119	0.87 (0.66-1.15)	0.76 (0.53-1.10)
Yes	31/39	80/52	15/23	1.82 (1.01-3.28)	0.82 (0.37-1.83)

^a^Total glioma patients including glioblastoma multiform, astrocytomas and other gliomas.

^b^Adjusted for age and gender.

### *APE1/Ref-1 *rs1760944 and risk of gliomas stratified by histology

Next, we performed the stratified analysis for *APE1/Ref-1 *rs1760944 by glioma histology. As shown in Table 4, statistically significant differences were observed in allele and genotype distributions of rs1760944 T/G between glioblastoma patients and control subjects (*P *= 0.032 and 0.041, respectively). Overall, the variant G allele was associated with a decreased risk of glioblastoma compared with the T allele (OR = 0.80, 95% CI = 0.65-0.98, *P *= 0.032). Individuals with the homozygous -141GG genotype exhibited 46% reduced risk of glioblastoma (adjusted OR = 0.54, 95% CI = 0.34-0.87, *P *= 0.012), compared with the TT homozygote. According to the criteria suggested by Wacholder et al [32], this result remained robust given the appropriate prior probability of 25% (FPRP = 0.052) and 10% (FPRP = 0.140), but not with a prior probability of 1% (FPRP = 0.643). The *P-*associated with the trend test was 0.014 (*P *for trend = 0.014). However, no significant association of rs1760944 with risk of low-grade astrocytomas (astrocytomas except for glioblastoma) and other gliomas was found in this study. As the number of glioblastoma subjects was not large enough for stratification analysis, we did not further evaluate the association between the rs1760944 T/G and risk for glioblastoma by age, gender, smoking status and family history of cancer.

**Table 4:** Associations between *APE1/Ref-1 *rs1760944 and risk of different histological types of gliomas

rs1760944	Controls (824)	Glioblastoma (241)				Astrocytomas (284)				Other gliomas (241)			
						
	No. (%)	No. (%)	*P*^a^	OR (95% CI) ^b^	*P*^c^	No. (%)	*P*^a^	OR (95% CI) ^b^	*P*^c^	No. (%)	*P*^a^	OR (95% CI) ^b^	*P*^c^
Alleles													
T	898 (55.0)	286 (60.6)	**0.032**	1.00 (reference)	**0.032**	309 (55.6)	0.821	1.00 (reference)	0.821	263 (55.7)	0.783	1.00 (reference)	0.783
G	734 (45.0)	186 (39.4)		**0.80 (0.65-0.98)**		247 (44.4)		0.98 (0.81-1.19)		209 (44.3)		0.97 (0.79-1.19)	
Genotypes													
TT	237 (29.0)	81 (34.3)	**0.041**	1.00 (reference)		86 (30.9)	0.634	1.00 (reference)		66 (28.0)	0.592	1.00 (reference)	
TG	424 (52.0)	124 (52.6)		0.84 (0.60-1.17)	0.298	137 (49.3)		0.87(0.64-1.19)	0.390	131(55.5)		1.11(0.79-1.56)	0.539
GG	155 (19.0)	31 (13.1)		**0.54 (0.34-0.87)**	**0.012**	55 (19.8)		0.99(0.66-1.47)	0.950	39 (16.5)		0.91(0.58-1.43)	0.686
			*P*^d ^for trend = **0.014**			*P*^d ^for trend = 0.832				*P*^d ^for trend = 0.813			

^a^Allele and genotype frequencies in cases and controls were compared using χ^2 ^test.

^b^Allele-specific ORs were not adjusted; genotype-specific ORs were adjusted for age and gender.

^c^*P *value from unconditional logistic regression analyses.

^*d*^Trend tests were calculated using the genotype as an ordinal variable in the regression model.

### Post-hoc power calculation

We also performed post-hoc power calculation based on the observed variant allele frequencies in both control and case groups, using post hoc power analyses of G*Power3 (http://www.psycho.uni-duesseldorf.de/abteilungen/aap/gpower3). It showed that with a sample size 1052 (236 glioblastoma cases and 816 controls), the study had > 90% power in detecting associations of rs1760944 polymorphism with risk of glioblastoma, at a significance level of 0.05 (*df *= 2).

## Discussion

In this case-control study, we investigated the role of a -141T/G variant (rs1760944) in the *APE1/Ref-1 *promoter in 766 glioma patients and 824 cancer-free controls. We did not observe that rs1760944 T/G SNP was significantly associated with all gliomas. However, our findings indicated a protective effect of rs1760944 GG genotype in glioblastoma. To our knowledge, this is the first genetic epidemiological study on the association between the *APE1/Ref-1 *-141T/G polymorphism and risk of gliomas. These findings suggested that the variant *APE1/Ref-1 *-141GG genotype may not play a major role in the etiology of gliomas but may contribute to a subtype of gliomas.

Recently, two independent research groups have reported the association between the *APE1/Ref-1 *-141T/G polymorphism and lung cancer risk [28,29]. Lo et al. [28] found that the variant *APE1/Ref-1 *-141TG or GG genotype was associated with a significantly decreased risk compared with TT homozygote in a hospital-based case-control study including 730 lung cancer cases and 730 cancer-free controls in Taiwan. Furthermore, the decreased risk was observed among the heavy smokers, but not among the light or never smokers, which reflected a gene-smoking interaction in the development of lung cancer. In two studies comprising a total of 1072 lung cancer patients and 1064 cancer-free controls in Chinese populations, Lu et al. [29] at nearly the same time reported that the similar protective effect of the *APE1/Ref-1 *-141GG genotype against lung cancer risk, particularly among old subjects, current smokers, and subjects with a positive family history of cancer in their first-degree relatives. These data suggested that these variables may modify the effect of the -141T/G polymorphism on lung cancer. However, such gene-environment interactions were not observed in our study. Given that smoking is not associated with gliomas [2], one would not necessarily expect any effect modification of the SNP effect by smoking.

In this current study, the genetic effect of -141GG genotype was observed for glioblastoma (WHO grade IV), but not for other gliomas. Our interpretation of the results is that different glioma subtypes or grades may be potentially involved with variable molecular and genetic events. Similar observations have been reported by Shete et al. [33] and Simon et al. [34]. These findings reflected the possible existence of differential genetic susceptibility profiles for different glioma subtypes. Our results suggested that the *APE1 *may be a possible marker of susceptibility to glioblastoma *vs. *other gliomas. On the other hand, the preliminary finding may be attained through chance alone since the observed association was not noteworthy (FPRP = 0.643) assuming a prior of 1%.

*APE1/Ref-1 *has been implicated in the development and progression of various cancers [35-38]. A previous study has demonstrated that substantial elevation of AP endo activity, as well as APE/*Ref-1 *levels, is characteristic of adult gliomas [38]. And AP endo activity is significantly greater in high-grade than in low-grade tumors. Therefore, while the functional role of APE1/Ref-1 in gliomas is not completely understood, a biological relevance in glioblastoma seems highly plausible. The *APE1*/*Ref-1 *-141T/G variant was located in the promoter of the gene, only 141-bp upstream from the transcription initiation site. It is known that the promoter and 3'UTR regions of a gene have functions in regulating transcription, mRNA stability, and gene production [39]. Variants in the promoter region may function by modulating gene transcription and leading to abnormal protein expression [40,41]. The functional effect of the *APE1*/*Ref-1 *-141T/G polymorphism has been reported in lung cancer [28,29]. In this study, our results do not disclose whether -141T/G polymorphism is correlated with *APE/Ref-1 *expression level or functions in glioblastom. Thus, more extensive studies are needed to resolve the functions of the -141T/G polymorphism. Nevertheless, we cannot at this time rule out the possibility that the genetic interactions between *APE1/Ref-1 *variants and other DNA repair genes are involved in glioblastoma.

Previous studies on the association between *APE1*/*Ref-1 *polymorphisms and risk of gliomas or its subtypes only evaluated the nonsynonymous SNP rs1130409 [19,22]; however, the findings were inconsistent. Liu et al [19] reported that the homozygous AA genotype of rs1130409 was associated with a reduced risk of gliomas in 373 glioma Caucasian cases and 365 Caucasian controls. The protective effect of rs1130409 was more evident in high-grade glioma (i.e. glioblastoma). However, the association of rs1130409 with glioblastoma was not replicated in a larger study including 1,015 glioblastoma cases and 1,994 controls [22]. The discrepancies in findings between two studies may reflect chance associations and/or differences in characteristics of the study populations. Considering our glioma sample size was not large, we did not examine rs1130409 in the current study. Many other studies have investigated the role of DNA repair genes in gliomas or its subtypes [42-47]. Unfortunately, few studies have been able to repeat the results of other studies. Recently, Liu et al [48] reported that some polymorphisms in DNA repair genes were associated with glioblastoma survival. However, the role of *APE1*/*Ref-1 *polymorphisms in glioma survival need to be further investigated.

Two recent genome-wide association (GWA) studies with risk of gliomas did not identify an association between the rs1760944 polymorphism and glioma risk [33,49]. The contradictory findings between GWA studies and our present study might be due to differences between populations or the different reporting criteria for a *P *value. Patients of GWA studies were whites from North America and European, and the allele and genotype frequencies described in the HapMap project for the rs1760944 polymorphism are different in CEU and CHB populations. However, these hypotheses can only be tested in GWA studies of gliomas in Chinese population.

Our study has several limitations. Mostly notably, the observed association in glioblastoma alone may be a subgroup finding due to chance. Therefore the finding requires validation in other independent cohorts. Secondly, our study did not provide any mechanistic information regarding glioma risk or protection. Specifically, we did not determine if the *AEP1*/*Ref-1 *promoter variant was correlated with the *AEP1*/*Ref-1 *expression or its protein activity. Third, we only evaluated one SNP in the *AEP1*/*Ref-1 *gene, which was not sufficient to systematically evaluate glioma risk for the studied gene. It should be pointed out that our observed association could be due to linkage disequilibrium with the true causal SNP in or outside the *AEP1*/*Ref-1 *gene. In addition, we did not evaluate the gene-environment interactions in glioblastoma since the number of glioblastoma patients was small. However, replication of the finding should be warranted before proceeding to the gene-environment studies.

## Conclusions

This study suggests a decreased risk of glioblastoma associated with a specific *APE1/Ref-1 *genotype in a Chinese Han population. Large-scale studies with ethnically diverse populations and functional evaluation are warranted to confirm our findings and to further elucidate the significance of the variant in glioblastoma.

## Competing interests

The authors declare that they have no competing interests.

## Authors' contributions

KZ collected the samples, performed the further interpretation of results, and drafted the manuscript. DH and JL carried out the data analysis generated the tables, as well as manuscript preparation. WF and CH participated in DNA sample preparation and co-worked in data analysis. GC also contributed to sample collection and face-to-face interview of each participant. RD and YM coordinated the study and participated in its design. QW and LZ and GD provided advices for preparing the manuscript and final editing of the manuscript. All authors read and approved the final manuscript.

## Pre-publication history

The pre-publication history for this paper can be accessed here:

http://www.biomedcentral.com/1471-2407/11/104/prepub
